# Supplementary material for: Are carbon emissions trading and green financial instruments synergistic? -Comprehensive quantitative research based on content analysis
Source: PLoS One. 2024 Mar 7;19(3):e0298601. doi: 10.1371/journal.pone.0298601 (PMC10919601; doi:10.1371/journal.pone.0298601)
Supplement: S1 Data — (ZIP) [file pone.0298601.s001.zip › Data/地级市Z_F工作报告.zip!/╔╜╢1⁄2/╤╠╠¿╩╨/└┤╫╘╣1⁄2╓┌║┼í╛┬φ┐╦╩2╛▌═°í┐_2012.docx]

各位代表：

现在，我代表市人民政府向大会报告工作，请予审议，并请市政协委员和其他列席会议的同志提出意见。

一、过去四年的政府工作

市十五届人大一次会议以来，全市上下在中共烟台市委的正确领导下，坚持以邓小平理论和“三个代表”重要思想为指导，以科学发展观为统领，深入贯彻落实党的十七大和十七届三中、四中、五中、六中全会精神，解放思想、抢抓机遇，攻坚克难、拼搏进取，较好地完成了市十五届人大历次会议确定的任务目标，实现了经济社会发展的新跨越。

———经济综合实力显著增强。四年来，全市生产总值连续迈上3000亿元、4000亿元台阶，2011年达到4906.8亿元，年均增长13.3%；地方财政收入达到303.2亿元，比2007年翻了一番多。

———改革开放取得丰硕成果。各领域改革纵深推进。2011年完成外贸进出口总额453.5亿美元，是2007年的1.9倍；四年累计实际使用外资46.4亿美元。

———城乡面貌发生明显变化。全市城镇化率达到57.2%，比2007年提高2.6个百分点。中心城市建成区面积由176平方公里扩展到265平方公里。农村面貌和生态环境不断改善。

———人民生活水平持续提高。城市居民人均可支配收入和农民人均纯收入年均分别增长12.2%和13.8%。经过全市上下的共同努力，烟台成功获得全国文明城市“三连冠”、社会治安综合治理优秀城市“五连冠”，主要指标达到或基本达到全面小康社会标准，广大市民的生活幸福感和城市荣誉感显著增强。

四年来，重点抓了以下工作：

(一)有效应对国际金融危机冲击，保持经济平稳较快增长。认真贯彻宏观调控政策，制定实施了40多项扩内需、保增长措施。争取中央和省资金93.5亿元，实施单体投资过2亿元的市级重点项目453个。四年累计完成固定资产投资9774亿元，年均增长23.6%。全面落实鼓励消费的各项政策，扩大家电、汽车、摩托车下乡规模，培育旅游、文化等消费热点。2011年，社会消费品零售总额达到1616.5亿元，年均增长18.1%。加强物价监测预警，抓好“菜篮子”工程建设，完善农副产品流通体系，物价保持基本稳定。坚决落实国家房地产调控政策，房地产市场实现平稳健康发展。狠抓节能减排，累计关停28万千瓦小火电机组、77条立窑水泥生产线，圆满完成省下达的节能减排目标任务。深入实施山东半岛蓝色经济区、黄河三角洲高效生态经济区、胶东半岛高端产业聚集区建设“三大战略”。2011年，全市完成海洋产业产值1480亿元，年均增长16.2%。

(二)调整优化产业结构，不断完善现代产业体系。三次产业比例由2007年的8.3：61：30.7调整为目前的7.4：57.7：34.9。加快工业调整振兴步伐。大力实施“3·50”和“8515”工程，产业集群快速扩张，骨干企业不断壮大。规模以上工业主营业务收入在全省率先突破1万亿元，2011年达到12086亿元，利润、利税年均分别增长24.1%和23.2%，主营业务收入过百亿元企业由2007年的6家增加到现在的16家。新增中国驰名商标37件、山东名牌产品58个。海阳核电、万华工业园、张裕工业园等一批重大项目启动建设。大力推进科技进步和自主创新，新增国家级工程技术研究中心、企业重点实验室、企业技术中心11家。累计授权专利1.3万余件，取得科技成果788项，获国家科学技术奖15项，引进各类人才近10万人。入选国家创新型试点城市，进入全国科技进步先进市、国家知识产权工作示范城市行列。高新技术产业产值占规模以上工业比重达到38.7%。繁荣发展现代服务业。2011年，全市服务业增加值达到1714.5亿元，年均增长16.5%；接待海内外游客3918万人次，实现旅游总收入402.5亿元；港口货物吞吐量达到2.4亿吨，集装箱吞吐量达到170.8万标箱；航空旅客吞吐量和货邮吞吐量分别达到255万人次和4.1万吨。成功举办了国际葡萄酒节和果蔬·食品博览会等一系列重要节会。新引进39家金融机构。2011年末，金融机构本外币存、贷款余额分别达到4468亿元和3071亿元。巩固发展现代农业。累计发放各类涉农补贴13.5亿元，粮食连续九年丰收，新增22件国家地理标志证明商标和4个国家地理标志保护产品。烟台荣获中国食品名城、中国绿色食品城称号。

(三)深化改革扩大开放，进一步增强发展活力。市县乡三级政府机构改革、集体林权制度主体改革基本完成。财政体制改革、文化体制改革、农村产权制度改革、小城镇综合配套改革稳步推进。医药卫生体制改革完成阶段性任务。国有企业改革不断深入，市管企业资产总量比2007年扩大1.5倍。民营经济增加值、上缴税金分别占全市总量的80%和60%以上。新增上市公司14家，全市上市公司四年累计融资195亿元。大力实施“三个引进”，新落户世界500强投资项目22个，全市涉外税收占全部税收的比重达到55.7%。着力转变外贸增长方式，机电产品和高新技术产品出口比重分别达到67.6%和32.6%。加快“走出去”步伐，累计实施境外投资项目124个。外事侨务工作取得新成绩，对外经济文化交流日益活跃。经济技术开发区综合实力在国家级园区排序中跃居第6位，对全市的贡献份额进一步扩大。保税港区获批成立并封关运作，烟台高新区、招远开发区晋升为国家级园区。

(四)积极实施城镇化战略，努力提高城乡统筹发展水平。编制完成了城市总体规划、海岸带规划和相关专项规划。中心城市累计投入城建资金500多亿元，高标准建成市文化中心、新火车站等标志性建筑，新建和改扩建红旗中路、北马路等主次干道40余条，打通黄金顶、璜山、通世南路隧道，启动实施毓璜顶医院周边、北马路区片、幸福区片等旧城旧村改造项目，大力推进大南山、夹河、辛安河开发整治和滨海一线景观建设。蓝烟铁路电气化改造、莱山国际机场改造等工程全面完成，烟台新机场、烟台港西港区、青烟威荣城际铁路等一批重点工程开工建设，烟台至海阳高速公路主体工程进展顺利，累计新建改建干线公路384公里、农村公路2394公里。全市新建扩建污水处理厂8座，改扩建生活垃圾处理场4座，4300多个村(居)实现城乡垃圾一体化处理。市区新增集中供热面积781万平方米、天然气用户29.3万户。加大城乡环境综合整治力度，数字化城市管理实现市区全覆盖。莱州、莱阳、龙口、海阳四市跨入中等城市行列，蓬莱市被国务院命名为国家历史文化名城。全市有20个镇(街)成为国家重点镇和省中心镇，沙河镇成为省级扩权强镇试点镇，1600多个村庄达到星级示范村标准。全市新建农房29万套，改造危房5万户，超额完成农村住房建设和危房改造三年任务。完成了16座大中型水库和559座小型水库除险加固任务，胶东引黄调水干线主体工程基本完工，农村自来水普及率达到96%，发展节水灌溉106万亩。新增造林绿化面积80万亩，森林覆盖率达到40%。昆嵛山保护区升级为国家级自然保护区。

(五)着力保障和改善民生，促进社会事业全面进步。每年实施为民服务实事工程，解决了一批群众普遍关心的热点难点问题。突出抓好大学生、城镇就业困难人员和农民工等重点人群就业，累计新增城镇就业65万人、农村劳动力转移就业14.5万人。2011年末，城镇登记失业率控制在3.25%。城镇居民养老保险、基本医疗保险和新农保、新农合实现全覆盖，企业职工五项社会保险实现市级统筹，企业退休人员基本养老金、老年农民养老待遇、城乡低保和农村五保标准逐年提高。新建和筹集保障性住房2.7万余套。新建扩建了市老年福利服务中心、儿童福利院、残疾人托养服务中心和乡镇敬老院等一批公共服务设施。发放爱心捐款2.5亿元，救助困难群众和学生近80万人次。全面实施中小学校舍安全、校园安保、仪器更新等教育惠民工程，在全国、全省率先全部免除城乡义务教育阶段杂费和课本费。县乡村三级医疗卫生服务网络更加完善，基本公共卫生服务覆盖城乡居民。健全完善公共文化服务体系，大剧院、青少年宫、群众艺术馆、博物馆新馆等一批文化设施相继投入使用。体育事业蓬勃发展，烟台籍运动员在北京奥运会、广州亚运会、十一届全运会等重大赛事上屡创佳绩。第三届亚洲沙滩运动会场馆及配套工程建设基本竣工，各项筹备工作顺利推进。积极做好国防教育与动员、国防后备力量、人民防空和双拥共建工作，军政军民团结更加巩固。人口和计划生育工作连续四年获得全省第一名，气象地震工作在防灾减灾中发挥了重要作用，老龄、妇女、儿童、红十字、残疾人和慈善事业得到加强，广播电视、新闻出版、史志档案、民族宗教等工作取得新成绩。

(六)加强民主法制和精神文明建设，维护和谐稳定的良好局面。坚持科学民主决策，推进依法行政，完善基层群众自治机制，深化行政审批制度改革，政府公信力、执行力不断增强。认真执行市人大及其常委会的决议、决定，自觉接受市人大、政协和社会各界监督。“五五”普法和依法治理工作取得实效。全面加强政府廉政建设，不断强化财政监督和审计监督。广泛开展创先争优、群众性精神文明创建活动，涌现出全国道德模范李登海、大学生村官张广秀、优秀供电所长汪浩、“最美女孩”刁娜、“爱心老人”刘盛兰、消防英雄孙茂珲等一批先进典型。深入推进和谐稳定模范城市建设，加强和创新社会管理，重视做好信访稳定工作，应急管理体系更加完善，食品药品安全监管力度加大，安全生产形势保持稳定。全力以赴支援汶川特大地震、青海玉树地震、甘肃舟曲泥石流等自然灾害抢险救灾工作，提前圆满完成对口援建北川任务。帮扶栖霞、支持长岛和对口支援工作取得新成果。

各位代表！过去四年极不平凡。全市人民共同经历了国际金融危机的严重冲击，经受了抗震救灾和援建灾区的重大考验，战胜了各种自然灾害的严峻挑战，成绩来之不易。这些成绩的取得，是党中央、国务院，省委、省政府和中共烟台市委正确领导的结果，得益于历届政府打下的坚实基础，离不开市人大、市政协、各民主党派和社会各界的监督支持，凝聚着全市人民的智慧、心血和汗水。在此，我代表市人民政府，向651万烟台人民，向市人大代表、政协委员，向各民主党派、工商联、各人民团体和社会各界人士，向中央省属驻烟单位、驻烟中国人民解放军指战员、武警部队官兵，向所有关心支持烟台发展的港澳同胞、台湾同胞、海外侨胞、国际友人，表示衷心的感谢，并致以崇高的敬意！

各位代表！四年来的丰富实践和磨砺锻炼，使我们进一步认识到：坚持党的领导、保持政令畅通，认真贯彻执行上级党委、政府的各项决策部署，团结带领全市人民努力奋斗，是我们推动事业从胜利走向胜利的根本保证；坚持科学发展、加快发展，始终把发展作为第一要务，转变发展方式，调整经济结构，是我们实现富民强市的必由之路；坚持解放思想、提升境界，始终在“三个坐标”中高点定位，对照先进城市查找不足，永不满足，永不懈怠，是我们不断开创新局面的重要法宝；坚持改革开放、锐意创新，牢牢把握发展机遇，创造性地开展工作，是我们打造竞争新优势的强大动力；坚持求真务实、真抓实干，始终保持奋发有为的精神状态，脚踏实地，拼搏进取，是我们完成工作目标的坚实基础；坚持以人为本、统筹城乡，着力保障和改善民生，让发展成果更多更好地惠及群众，是我们做好一切工作的最终目的。这些经验总结是我们战胜各种困难、推动跨越发展的宝贵财富，我们一定要倍加珍惜，并在不断丰富完善中发扬光大。

在总结成绩的同时，我们也清醒地看到：经济运行面临着复杂形势，保持平稳较快发展压力增大；服务业发展相对滞后，自主创新能力还不够强，节能减排、环境保护任务艰巨，转变发展方式任重道远；外经贸形势依然严峻，进一步扩大开放难度加大；部分企业生产经营困难，铁路交通、土地、资金等瓶颈制约较为突出；民生投入仍需给力，城乡居民收入有待提高，一些群众集中反映的热点难点问题还没有得到很好解决；政府自身建设还要加强，积极进取、主动服务、大胆改革、提速增效等方面做得还不到位，个别领域甚至出现腐败现象。对于这些问题，我们将采取有效措施，认真加以解决。

二、今后五年的奋斗目标

今后五年是烟台转方式调结构、加快推进现代化建设的关键时期。综观国内外形势，世界经济复苏的不稳定、不确定性上升，国内经济发展中不平衡、不协调、不可持续的问题依然突出。但同时也要看到，我国仍处于重要的战略机遇期,经济长期向好的大趋势没有改变，我省“三大战略”的实施为烟台带来了重大发展契机。经过长期不懈奋斗，烟台已经打下了较为坚实雄厚的产业基础，积累了许多战胜困难的宝贵经验，具备了努力再上台阶的实力条件，形成了奋发有为、干事创业的浓厚氛围。只要我们以科学的思维和超前的战略眼光谋划未来，以高度的责任感和强烈的忧患意识抢抓机遇，以坚定的信心和顽强的拼搏精神应对挑战，以扎实的作风和高昂的工作热情创造业绩,就一定能够开启全市又好又快发展的新篇章！

刚刚闭幕的中共烟台市第十二次党代会确立了烟台未来发展的总体思路，描绘了今后五年的美好蓝图。按照市党代会的战略部署，今后五年政府工作的指导思想是：高举中国特色社会主义伟大旗帜，以邓小平理论和“三个代表”重要思想为指导，深入贯彻落实科学发展观，牢牢把握科学发展主题和加快转变经济发展方式主线，紧紧围绕“一个率先、三个坐标、三个跨越”的奋斗目标，积极调整经济结构，纵深推进改革开放，着力抓好自主创新和节能减排，统筹推动城乡发展，切实保障和改善民生，保持经济平稳较快发展和社会和谐稳定，巩固提升全面建设小康社会成果，率先基本实现现代化。

———注重发挥优势、加快转调，努力把烟台建设成为综合实力大幅提升、发展基础更加牢固的城市。充分发挥“三大战略”政策叠加优势，集中培育蓝色经济、特色经济、城市经济、县域经济和民营经济，大力发展先进制造业、现代服务业和安全优质高效农业。牢牢把握发展实体经济这一坚实基础，进一步营造勤劳致富、开放创新、诚信和谐的社会氛围。到2016年，全市生产总值突破8700亿元，年均增长12%左右；地方财政收入突破600亿元，比2011年翻一番；三次产业比例调整为4.5：53.5：42。

———注重以人为本、改善民生，努力把烟台建设成为群众幸福感普遍增强、社会更加和谐稳定的城市。坚持把保障和改善民生作为政府工作的根本出发点和落脚点，全面发展各项社会事业，努力提高人民生活水平，让困难群众享有更多实惠，让老年人得到更多关怀，让青年人拥有更多机会，让孩子们获得更多关爱，让全市人民共享幸福和谐。到2016年，城市居民人均可支配收入和农民人均纯收入年均增幅均达到12%以上。

———注重改革创新、科技进步，努力把烟台建设成为创新能力显著提升、体制机制更加科学的城市。大力实施科教兴烟、人才强市和知识产权战略，鼓励科技进步与自主创新，着力培育高端高质高效产业、战略性新兴产业，加快信息化与工业化“两化”深度融合。积极推进经济、文化、社会等各领域改革创新，建设国家创新型城市。到2016年，全市高新技术产业产值占规模以上工业比重提高到43%。

———注重开放引领、内需驱动，努力把烟台建设成为开放水平不断跃升、市场经济更加繁荣的城市。强化对外开放在兴市、强市中的重要地位，坚持“引进来”与“走出去”并举，争取建立中日韩自由贸易先行试点区。牢牢把握扩大内需这一战略基点，千方百计促进城乡消费，增强消费、投资、出口对经济增长的协同拉动。到2016年，全市进出口总额达到730亿美元，五年累计实际使用外资60亿美元；社会消费品零售总额、固定资产投资年均增幅均保持在16%左右。

———注重统筹城乡、保护生态，努力把烟台建设成为城乡面貌显著改善、环境质量更加优良的城市。积极稳妥推进城镇化，创建生态文明综合示范区，加快社会主义新农村建设，推动基本公共服务和基础设施更多地向农村延伸，构建城乡一体化发展新格局。牢固树立绿色、低碳、节能理念，高度重视耕地保护和集约节约用地，使资源消耗更小、环境代价更低、生态质量更优，建设资源节约型、环境友好型社会。到2016年，全市城镇化率达到64%以上；单位生产总值能耗和主要污染物排放显著下降；森林覆盖率达到42%。

———注重内涵挖潜、提升品位，努力把烟台建设成为文化软实力持续提高、社会更加文明进步的城市。坚持先进文化的前进方向，加快完善公共文化服务体系，推进文化体制机制创新，积极发展公益性文化事业，做大做强文化产业，深入发掘特色文化，搞好历史文化遗产的保护与利用，打造历史文化名城。推进社会主义核心价值体系建设,不断提高全体市民的思想道德素质,让崇高的道德文化深入广大市民心灵,转化为强大的社会正义力量。一以贯之地加强文明城市建设，让文明在传承中积淀，在积淀中丰富，在丰富中升华，把全国文明城市的荣誉永留烟台！

未来五年，我们将举全市之力抓好五项重点工作：

(一)推动重要板块加快崛起，形成充满活力的新的经济增长极。坚持“蓝区”统领，“蓝”、“黄”、“高”结合，统筹推进实施“三大战略”，构建“一极领先、多极崛起”的发展格局。“一极领先”：依托牟平区、高新区、莱山区、芝罘区、保税港区滨海区域和相关岛屿，规划建设594平方公里的高技术海洋经济新区，打造蓝色经济东部增长极。“多极崛起”：以莱州湾为中心，发挥“蓝”、“黄”两大战略优势，加快莱州临港产业区建设，打造西部经济增长极。以丁字湾为中心,推动以海阳、莱阳为主体的海洋文化旅游产业聚集区建设，打造南部经济增长极。以龙口湾为中心，加快实施集中集约用海工程，建设龙口临港高端制造业聚集区、招远滨海科技产业园和蓬莱西海岸文化新区，打造北部经济增长极。搞好“蓬(蓬莱)—长(长岛)—龙(龙口)”旅游度假区规划建设，形成独具特色、海陆统筹、资源共享、优势互补的经济一体化协作区。在市域中部，依托芝罘、福山、莱山、开发区，大力实施旧城区、旧村居改造，完善市政基础设施，繁荣发展城市经济、蓝色产业，增强区域辐射带动功能；有效保护和有序开发以栖霞为主体的中部生态涵养区，建好烟台的生态屏障和永续水源地；高标准规划建设昆嵛山保护区，打造生态旅游与高端休闲养生目的地。经过五年的努力，烟台将形成“东西南北中”竞相发展、亮点纷呈的经济新格局，区域间发展的差异性、互补性、协调性显著增强。

(二)狠抓大产业大企业大项目，筑牢实体经济增长基础。加大“招大引强”、“三个引进”力度，着力培育千亿产业集群、千亿企业集团、百亿龙头企业和十亿骨干企业“四个方阵”。未来五年，万华工业园、张裕工业园、东岳汽车三期项目将建成投产，总部经济基地、服务外包示范园区将建成运营，海阳核电、华电国际莱州电厂实现并网发电。到2016年，力争培育4-6个产值3000至5000亿元的支柱产业、8个产值过千亿元的产品集群、5个产值过千亿元的企业集团、20个以上产值过百亿元的龙头企业，形成100万辆整车、1000万台电脑、4000万部手机的年生产能力。

(三)构建立体交通运输体系，建设面向东北亚的交通物流枢纽城市。加快推进烟台新机场、烟台港西港区、青烟威荣城际铁路、龙烟铁路建设，力争在未来2-3年陆续投入使用。适时启动龙口至青岛、蓬莱至栖霞高速公路和城区快速路建设，规划筹建部分城市轨道交通。拓展与韩国港口城市的陆海汽车联运业务，积极推动中韩铁路轮渡项目。到2016年，新增铁路干线250公里以上，全市港口货物吞吐量力争突破3.5亿吨，航空旅客吞吐量争取达到500万人次。

(四)精心打造中心城区亮点景观，塑造人文与自然交融、宜居宜业的滨海城市新形象。启动实施北马路滨海区片整体改造，努力使芝罘湾成为代表烟台城市形象的蓝色休闲港湾；加快推进大南山生态景区建设，打造集生态、休闲、观光于一体的城市山体；有序推动养马岛、崆峒岛、芝罘岛保护性开发，建设风光秀美的魅力海岛；继续实施夹河、辛安河、鱼鸟河、沁水河治理和沿岸整治改造，形成贯通山海、融合城区的滨河景观带；适时启动烟台山、朝阳街、所城里区片开发保护，塑造彰显烟台历史文化特色的建筑群落。五年后的烟台，将以山、海、岛、河、港、街的点线连结，更加清晰地凸显城市的形象美、风景美、生态美和人文美。

(五)以更大的力度惠民富民，不断提高广大市民的幸福指数。大力实施收入同步提升计划，实现居民收入增长与经济发展同步，劳动报酬增长与劳动生产力提高同步。继续搞好保障性安居工程建设，稳妥推进农村新型社区建设，五年内新建和筹集保障性住房2.5万套以上。实施更加积极的就业政策，城镇登记失业率控制在3.5%以内。建立更高水平的社会保障体系，城乡救助机制更加完善。实施“蓝天、碧水、青山、绿地”四大生态工程，防治大气、水体、重金属、农业面源污染，推进工程减排、结构减排和管理减排，努力恢复植被、绿化山体，腾出更多的城市空间建设公园和绿地，让烟台的天更蓝、水更秀、山更绿、空气更清新。

各位代表！率先基本实现现代化是市委、市政府站在战略和全局的高度，对烟台未来五年发展提出的总目标。这一目标符合中央对东部地区率先发展的要求，适应烟台在全省及沿海开放城市中的发展地位，顺应烟台人民群众过上更好生活的新期待。我们将紧紧围绕这一目标，探索走出一条符合时代特征、具有烟台特色的基本现代化之路。经过五年的奋斗，全市人均生产总值达到2万美元以上，产业体系得到明显优化，产业结构更加科学合理，工业化程度接近先进制造业国家水平，科技创新能力达到国际公认的创新型城市标准，城镇化率、生态质量、社会保障、居民生活、社会管理达到或接近中等发达国家水平。五年后，一个风景美丽、生活美好、开放包容、文明富庶的烟台，将以崭新的面貌蓬勃崛起。我们坚信，有科学发展观的正确指引，有中共烟台市委的坚强领导，在全市人民的共同努力下，我们一定能够完成率先基本实现现代化的宏伟目标，再创烟台科学发展新的更大辉煌！

三、2012年的工作安排

今年是实施“十二五”规划承上启下的重要一年，我们党将召开十八大。做好今年的政府工作，意义十分重大。新的一年，我们要全面贯彻“稳中求进”的工作总基调，既要做到稳，更要追求进，要在进中求转调、进中求创新、进中求质量、进中求效益，以奋发有为的精神、攻坚克难的干劲和求真务实的作风，奋力开创政府工作新局面。

今年经济社会发展的主要预期目标是：全市生产总值增长11.5%；地方财政收入增长15%；全社会固定资产投资增长18%；社会消费品零售总额增长16%；实际使用外资保持稳定增长，外贸进出口总额增长10%；城市居民人均可支配收入和农民人均纯收入均增长12%；城镇登记失业率控制在3.5%以内；居民消费价格涨幅控制在4%左右；人口自然增长率控制在2‰以内；全面完成省下达的节能减排任务目标。

(一)着力推动经济平稳较快发展

扩大城乡居民消费。更多地增加城乡居民特别是困难群众收入，提高社会消费能力。完善消费政策，培育消费热点，拓宽消费渠道，促进旅游、文化、家政等服务消费。构建现代商贸流通体系，抓好“万村千乡市场工程”和“放心粮油工程”，加快新天地都市广场、中粮大悦城等一批重点商贸项目建设，完成市区35处农贸市场提升改造工程。整顿规范市场秩序，让群众安全消费、舒心消费。年内，全市社会消费品零售总额预期完成1875亿元。

保持投资合理增长。优化投资结构，加大重点领域投资力度，集中推进195个市级重点项目建设。年内，完成全社会固定资产投资3400亿元。加大项目储备力度，策划论证一批对区域发展具有引领支撑作用的大项目、好项目。

保持物价总水平基本稳定。强化市场物价监管，加强主要农产品生产、收储和调运，抓好“菜篮子”工程、“早餐示范工程”和副食品基地建设，完善粮油市场保供稳价机制，推进“农超对接”和“区超对接”。健全社会救助保障标准与物价上涨挂钩联动机制，保障困难群众的基本生活。

促进房地产市场健康发展。严格落实国家房地产调控政策，引导市场有序开发、理性消费。调整优化住房供应结构，加快保障性安居工程建设，适度发展产业地产。年内，建设和筹集保障性住房6400余套。

强化财税金融保障。加强财源建设，抓好税收征管和非税收入管理，进一步提高税收占地方财政收入的比重。优化财政支出结构，优先保证民生改善、重要领域改革和重大项目建设投入。优化对金融机构的服务与协调，推动金融管理体制创新，鼓励金融机构增加对重点产业、薄弱环节和优质中小企业的信贷支持。加强金融监管和市场体系建设，加快组建农村商业银行，积极引进各类金融机构，有序发展村镇银行和小额贷款组织。推动企业境内外上市，设立市级创业投资引导基金，支持企业设立私募股权基金和基金管理公司。年内，新发展一批融资性担保公司和创业投资企业，金融机构贷款余额争取达到3470亿元。

(二)深入推进经济结构调整

推动工业优化升级。按照“高端化、集群化、信息化”的发展思路，提升发展机械、电子、食品、黄金、现代化工五大传统支柱产业，加快发展新能源和节能环保、新材料、生物技术、高端装备制造、新一代信息技术五大战略性新兴产业，膨胀壮大汽车、电脑、手机、葡萄酒等十大产品集群，在更高层次、更广领域推动工业加快发展。大力推进总投资500亿元的十个工业重点技术改造项目，抓好海阳核电一期、万华工业园、张裕工业园、中国北车风电装备制造等项目建设，推进通用东岳新增30万辆轿车、华电国际莱州电厂1、2号机组、台海玛努尔核电二期、神宇大功率柴油发动机等项目投产。年内，规模以上工业主营业务收入预期达到13000亿元。深入实施“8515”培强做大骨干企业工程，年内主营业务收入过10亿元的企业达到130户以上。

推动现代服务业提质增量。坚持“集聚化、规模化、特色化”的主攻方向，落实各项扶持政策，推动服务业跨越发展。把旅游业作为支柱产业来培育，整合开发滨海一线旅游资源，加快大南山生态园福临夼景区主体工程、五彩文化广场二期等重点项目建设，抓好9个省级旅游度假区建设，推动海阳、蓬莱争创国家级旅游度假区，大力发展生态乡村旅游，搞好城市整体形象筹划和推介。年内，全市接待海内外游客预期达到4500万人次。大力发展现代物流业，加快推进综合物流园区、大型批发市场等重点项目。深入实施“以港兴市”战略，加快建设东北亚枢纽大港和区域性国际航运物流中心。年内，全市港口货物吞吐量达到2.6亿吨。提升发展商务服务业，加快滨海广场和迎春大街商务聚集区建设，抓好雨岱山渔人码头、鲁菜文化一条街等特色街区培育，高标准办好第六届烟台国际葡萄酒博览会和第十三届国际果蔬?食品博览会。超前谋划邮轮经济和通用航空产业。加大对文化创意、养生养老、社区服务等新兴服务业的培植力度。年内，服务业增加值占生产总值的比重提高1.4个百分点。

推动现代农业科学发展。加快农业“产业化、品牌化、标准化”进程，努力构建安全优质、特色高效的现代农业产业体系。落实强农惠农富农政策，实施“粮油高产创建”工程。调整优化农业结构，大力发展苹果、葡萄、海参等优质高效产业，加强地理标志农产品品牌保护。健全完善现代农业技术体系，提高农作物机械化水平。搞好重大动植物疫病防控，建立健全农产品质量安全监管和检验检测体系。鼓励发展农业专业合作社，纵深推进农业产业化，建好莱州国家级现代农业示范区和蓬莱农业高新技术产业示范区。加快构建现代水网工程体系，推进南水北调东线续建配套工程，实施10座大中型、70座小型水库除险加固工程。

提升科技创新水平。抓好国家创新型城市建设，实施科技创新“六个十”工程，支持30个重大科技项目研发。大力发展高新技术产业，力争高新技术企业达到180家以上。加强与大型国企、高校院所的合作，积极引进高层次科研机构。深化国家知识产权工作示范城市建设，提升全社会知识产权创造、保护、运用能力。加大“招才引智”力度，实施高端人才引进“双百计划”，加强人才队伍建设，努力形成以用育人、以用聚才的良好局面。

抓好节能环保工作。严格落实节能减排目标责任制，加强工业、建筑、交通、公共机构等领域用能管理，淘汰落后产能，推广节能新技术。大力发展循环经济，年内固体废物利用量达到880万吨。推进4个污水处理厂建设改造，加快电力行业脱硫、脱硝进程。积极实施四大生态工程，搞好农村面源污染防治，推进水系造林绿化及小流域综合治理，将PM2.5纳入常规空气质量监测体系。有效保护海洋，加强海域内污染源的监测和防控。年内，增加造林补植面积13.6万亩，综合治理水土流失面积160平方公里，新增2个国家级海洋保护区。

(三)全面提升特色经济发展水平

大力发展蓝色经济。落实并用好海洋产权交易中心、国家级海洋科研成果转化基地等蓝色经济“十大试点政策”。加快蓝区重点区域开发建设，统筹推进东部高技术海洋经济新区、西部莱州临港产业区、南部丁字湾新区、北部龙口湾经济区建设，启动实施长岛休闲度假岛总体规划。在中心城区，打造面向海洋的高端商务服务业、创新型制造业和高层次旅游观光聚集区。培育壮大海洋装备制造、海洋工程、海洋新能源、海洋生物、海洋休闲旅游、海洋渔业等产业，扶持发展中集来福士、大宇造船、杰瑞石油等一批重点企业和重大项目，构建特色鲜明的现代海洋产业体系。年内，全市海洋产业产值预期达到1720亿元，增长16%以上。

扶持发展民营经济。实施“中小微企业百千万成长计划”，进一步落实扶持中小微企业发展政策，逐步健全融资担保服务体系，促进其持续健康发展。年内，新发展个体工商户和小微型企业2.7万家以上，民营经济上缴税金增长12%。

提升发展园区经济。推动经济技术开发区跨越发展，年内生产总值突破千亿元，巩固提升在全市开放发展中的龙头地位。加快高新区基础设施建设，抓好产业项目布局，打造高端高质高效园区。支持保税港区探索建立科学高效的物流监管模式。扶持昆嵛山保护区在严格保护生态的基础上发展旅游、文化产业。鼓励招远经济开发区借势发展，争取更多省级园区进入全省30强。加快张江—烟台高科技示范园、日本现代产业园、台湾农民创业园等特色园区的引进与建设，进一步提升园区集聚效应。

加快发展县域经济。鼓励强市率先发展，支持中等市跨越发展，扶持困难县市赶超发展，促进各县市在全国、全省县域经济中位次前移。继续开展帮扶栖霞、支持长岛和对口支援工作。抓好经济较强镇行政管理体制改革试点，推进中心镇加快发展，打造刘家沟、院格庄、龙泉、金城等一批以葡萄、温泉、生态为特色的风情小镇。

(四)继续深化重要领域改革

深入推进国有企业改革，抓好蓝天开发公司、北极星控股公司等企业的改革改制，进一步加强国有资产监管，建立更加规范的现代企业制度。落实国家税制改革政策，促进企业平稳健康发展。深化农村综合改革，促进土地承包经营权有序流转，启动林业专业合作社建设，推动城乡水务一体化管理。深化行政管理体制改革，稳妥推进事业单位分类改革。巩固医药卫生体制改革成果，年内基本药物制度覆盖全市统一规划的村卫生室。积极推进投融资体制改革，加快组建和培育集融资、开发于一体的城市建设实体公司。

(五)全面扩大对外开放

提升招商引资层次。继续“招大引强”，突出港台、日韩、欧美、澳洲、东南亚等重点区域，瞄准世界500强和行业龙头，突出先进制造业和现代服务业，开展高层招商、专题招商，引进一批战略性大项目。推进富士康、乐金显示等一批外企增资扩建。年内，全市实际使用外资13.5亿美元。

保持外贸稳定增长。优化出口结构，巩固日韩市场，拓展欧美市场，开发东南亚市场，扩大自主产权、自主品牌产品出口，扶持培育100家重点出口企业、300家潜力出口企业和500家成长型出口企业。加强农产品、工业产品质量安全示范区建设，创建国家科技兴贸出口创新基地和外贸转型升级示范基地。落实鼓励进口政策，增加紧缺资源能源、先进技术装备、关键零部件进口。年内，实现外贸进出口总额500亿美元，其中出口295亿美元。

推进区域战略合作。积极承接日韩产业转移，加强国内经济合作。鼓励有条件的企业“走出去”发展，抓好万华、玲珑等企业境外并购和投资项目建设。

(六)积极推进城乡统筹发展

高标准做好城乡规划。编制完成新一轮城市总体规划及城市综合交通等专项规划，搞好中心城区、昆嵛山保护区等重点区域的控制性详细规划，实现市区建成区控规全覆盖。加强对沿海沿河、山体周边、主次干道沿线等重点地带的规划保护，严肃查处违法违章建设。

大力推进城乡一体化建设。按照“东拓、西联、南进、北展、中优”的思路，大力实施年度投资306亿元的175项市级城建重点项目。抓好芝罘南部滨河生态智慧新城、北部临港蓝色国际新城和福山南部水上新城建设，启动莱山南部城区开发和牟平金山湾旅游区项目。加快旧城旧村改造步伐，积极推进胜利、慎礼、北马路、白石、滨海大社区等48个区片改造和恒隆广场、华润万象城、蓝海国际等城市综合体项目。优化城区路网结构，推进大海阳路跨线桥、胜利南路南延隧道、轸大路等45项市政交通项目。完善区域大交通网络，完成烟台港西港区大型深水码头主体工程，启动建设烟台新机场航站区，加速建设青烟威荣城际铁路，开工建设龙烟铁路，确保烟台至海阳高速公路5月底前主线贯通。加快社会主义新农村建设步伐，深入实施生态文明乡村建设工程，抓好农村饮水安全、垃圾一体化处理、道路建设和电网改造，新建改建农村公路1570公里，完成800个村庄环境综合整治。

精细化管理城市。规范居民小区管理，综合整治城市出入口、海岸线、旧城区和城乡结合部等区域，加快马路市场“退路进厅”。加强市区道路交通组织管理，建设智能交通诱导系统，优化调整重点路段信号配时和路口渠化，扩大公交车专用车道试点范围，新建一批大型停车场、过街天桥和公交场站，努力缓解城市交通拥堵。实施生活垃圾综合湿解二期、老城区雨污分流等44项公用设施和园林绿化项目。整合城市公交资源，优化公交网线布局。探索实施数字化执法，拓展城管数字化应用领域，提高城市管理水平。

(七)切实保障和改善民生

稳定和扩大就业，认真落实就业扶持政策，年内开发公益性岗位4000个，帮助5000名“4050”人员和零就业家庭等困难群体实现就业。争创首批国家级创业型城市，完成就业指导服务中心和烟台大学生创业园建设。年内，新增城镇就业再就业12万人以上，农村劳动力转移就业2万人以上。连续第八年提高企业退休人员基本养老金标准，新农合和城镇居民医疗保险补助标准提高到每人每年240元，城镇职工和居民住院费用报销比例分别达到85%和70%。继续抓好农村危房改造，促进农房建设工作常态化。加快发展老龄事业，健全养老服务体系，新增机构养老床位4000张，新建社区老年人照料中心15处。发展慈善事业，开展爱心捐助活动，关心帮助孤儿和残疾人等弱势群体，努力保障困难群众基本生活。

(八)不断繁荣社会事业

加大教育投入力度，合理配置教育资源，深入实施素质教育，大力发展高等教育、职业教育和民办教育，扩大优质教育资源辐射范围，全面提升教育质量。加快推进镇(街)公办幼儿园建设，抓好烟台二中高中部、第一职业中专、艺术学校迁建及高级师范学校、中小学综合实践教育基地建设。狠抓校车、校舍、校园安全，年内实现全市农村中小学校车服务全覆盖。建立全国文明城市建设长效机制，开展文明县市、文明村镇和文明单位创建活动。推进全国公共文化服务示范区建设，鼓励文化精品原创，开展“专业艺术院团演出季”等文化活动。推进科技馆、档案馆、民俗博物馆和县级文化馆、图书馆等场馆建设，新建文化大院示范点300个，农家书屋200家，在全市范围内实现公共博物馆、图书馆、文化馆免费开放。建设“书香烟台”，营造全民读书的浓厚氛围。积极创建全国首批全民健身示范城市，提升竞技体育综合实力，努力打造“体育名城”。实施卫生强基工程，抓好县级医院、乡镇卫生院和社区卫生服务中心建设。完善毓璜顶医院综合楼配套设施，积极推进市妇幼保健院、烟台山医院周边改造，加快建设滨州医学院烟台附属医院、107医院新院。完善公共卫生服务体系，强化重点疫病监控，加强全科医生和乡村医生培训，为全市6-8岁儿童免费开展龋齿防治。稳定低生育水平，提高出生人口素质。实施妇女儿童发展纲要，抓好外事侨务、民族宗教、史志档案、仲裁和红十字工作。支持国防和军队建设，做好人民防空、双拥共建工作。

(九)努力维护社会和谐稳定

加强民主法制建设，抓好“六五”普法和依法治市规划实施，加大法治烟台创建力度，做好法律服务、法律援助和法制宣传等工作。推进社会管理创新“八大体系”建设，夯实社会管理的基层基础。强化和改进社区服务与管理，建立多元化新型社会管理和服务机制。扎实推进和谐稳定模范城市建设，防范和打击各类违法犯罪活动。健全大调解工作机制，进一步畅通信访渠道，从源头上减少和化解社会矛盾。坚持不懈抓好安全生产，深入开展重点领域安全生产专项整治，坚决遏制重特大事故发生。加快市级综合应急指挥信息系统建设，做好气象、地震监测预警和防灾减灾工作，提高保障公共安全和处置突发事件能力。加强食品药品安全监管，建设全国安全食品名城。

各位代表！2012年第三届亚沙会将在烟台海阳举办，这是烟台历史上承办的层次最高、影响力最强的国际赛事。我们将竭尽全力做好各项筹办和组织工作，努力举办一届有特色、高水平的体育盛会，让亚沙会因烟台和海阳而更加精彩，让烟台和海阳因亚沙会而更显魅力！

四、加强政府自身建设

(一)不断解放思想，增强科学发展本领。进一步提升思想境界，瞄准高目标，谋划新思路，推动大发展。加强学习型政府建设，用马克思主义中国化的最新成果武装头脑、指导实践，坚持学以致用、学用结合，增强工作的预见性、科学性、创造性。科学判断形势，加强战略谋划，扬长避短，趋利避害，提高应对复杂局面的能力。善于用改革的方法、创新的思路解决前进中的问题，不断推动观念创新、科技创新、管理创新、体制机制创新，创造性地开展各项工作。

(二)弘扬务实作风，提升政府执行能力。把狠抓落实作为政府工作的重要职责，大力发扬知难而进、迎难而上的拼搏精神，脚踏实地，埋头苦干，做到落实、落实、再落实，确保各项工作落到实处、取得实效。大兴调查研究之风，深入实际、深入基层、深入群众，了解真实情况，解决实际问题。牢固树立正确的政绩观，多做打基础、利长远、惠民生的事情，力戒形式主义、官僚主义，反对弄虚作假和作表面文章，创造经得起实践、群众和历史检验的业绩。

(三)坚持依法行政，加强法治政府建设。坚持向人大及其常委会报告工作和向政协通报情况，自觉接受人大法律监督和政协民主监督，重视群众监督和舆论监督，加强与各民主党派、工商联、无党派人士的联系，进一步做好人大代表建议和政协提案办理工作。全面贯彻《行政强制法》和《山东省行政程序规定》，健全科学民主决策机制，把公众参与、专家论证、合法性审查等作为决策的必经程序。坚持公正文明执法，规范行政裁量权，强化行政监督和问责，积极推进行政复议体制机制创新。转变政府职能，深化行政审批制度改革，继续推进政务公开，促进政府工作提速增效。

(四)践行为民宗旨，保持清正廉洁本色。秉承执政为民理念，真心实意为群众办实事、做好事、解难事，扎实做好为民服务12件实事，坚决兑现向广大市民作出的庄严承诺。充分尊重人民群众的主体地位，把群众的需求看得最重，把群众的利益置于最高，让群众生活得更加幸福、更有尊严。弘扬艰苦奋斗、勤俭节约的优良传统，取消不合理、不必要的行政开支，把有限的资源和财力更多地用到改善民生上。深入开展党风廉政建设和反腐败斗争，加强领导干部经济责任审计，坚决纠正损害群众利益的不正之风，以清廉为民的实际行动赢得群众的信任和支持。

各位代表！我们正站在继往开来的历史起点上，新的征程已经开启，新的目标催人奋进。让我们更加紧密地团结在以胡锦涛同志为总书记的党中央周围，高举中国特色社会主义伟大旗帜，深入贯彻落实科学发展观，在中共烟台市委的坚强领导下，解放思想、锐意进取，团结拼搏、真抓实干，向着率先基本实现现代化的宏伟目标奋勇前进，以更加优异的成绩迎接党的十八大胜利召开！
